# NR1 and NR3B Composed Intranuclear *N*-methyl-d-aspartate Receptor Complexes in Human Melanoma Cells

**DOI:** 10.3390/ijms19071929

**Published:** 2018-06-30

**Authors:** Tibor Hajdú, Tamás Juhász, Csilla Szűcs-Somogyi, Kálmán Rácz, Róza Zákány

**Affiliations:** 1 Faculty of Medicine, Department of Anatomy, Histology and Embryology, University of Debrecen, H-4032 Debrecen, Hungary; hajdu.tibor@anat.med.unideb.hu (T.H.); juhaszt@anat.med.unideb.hu (T.J.); somogyics@anat.med.unideb.hu (C.S.-S.); 2 Department of Forensic Medicine, University of Debrecen, Faculty of Medicine, H-4032 Debrecen, Hungary; raczkalman@hotmail.com

**Keywords:** melanoma, melanocyte, NMDAR, NLS, nuclear Ca^2+^-signalling

## Abstract

Heterotetrameric *N*-methyl-d-aspartate type glutamate receptors (NMDAR) are cationic channels primarily permeable for Ca^2+^. NR1 and NR3 subunits bind glycine, while NR2 subunits bind glutamate for full activation. As NR1 may contain a nuclear localization signal (NLS) that is recognized by importin-α, our aim was to investigate if NMDARs are expressed in the nuclei of melanocytes and melanoma cells. A detailed NMDAR subunit expression pattern was examined by RT-PCRs (reverse transcription followed by polymerase chain reaction), fractionated western blots and immunocytochemistry in human epidermal melanocytes and in human melanoma cell lines A2058, HT199, HT168M1, MEL35/0 and WM35. All kind of NMDAR subunits are expressed as mRNAs in melanocytes, as well as in melanoma cells, while NR2B protein remained undetectable in any cell type. Western blots proved the exclusive presence of NR1 and NR3B in nuclear fractions and immunocytochemistry confirmed NR1-NR3B colocalization inside the nuclei of all melanoma cells. The same phenomenon was not observed in melanocytes. Moreover, protein database analysis revealed a putative NLS in NR3B subunit. Our results support that unusual, NR1-NR3B composed NMDAR complexes are present in the nuclei of melanoma cells. This may indicate a new malignancy-related histopathological feature of melanoma cells and raises the possibility of a glycine-driven, NMDA-related nuclear Ca^2+^-signalling in these cells.

## 1. Introduction

Glutamate is the main excitatory neurotransmitter of the mammalian central nervous system (CNS) acting on a broad range of ionotropic (iGluR) and metabotropic glutamate receptors (mGluR). Roles of mGluRs in physiological functions [1] or tumorigenesis [2] of various peripheral tissues, including melanocytes and melanoma have been recognized, but less is known about iGluRs in these contexts. According to their pharmacological properties (ligand binding) and sequence analogy iGluRs are divided into four subtypes: *N*-methyl-d-aspartate (NMDA), alpha-amino-3-hydroxy-5-methyl-4-isoxazolepropionic acid (AMPA), kainate and δ type glutamate receptors [3]. *N*-methyl-d-aspartate type glutamate receptors (NMDAR) are nonselective cation channels, with high permeability for Ca^2+^ [4] and require glycine coagonist for full activation. NMDARs are mostly known for playing role in synaptic plasticity, memory and learning [5]. NMDARs assemble as di- or triheterotetrameric complexes comprising three types of subunits (NR1, NR2 and NR3) with each subunit having several isoforms and in certain cases, multiple splice variants [6]. Two NR1 subunits possessing glycine binding sites are essential for channel formation, whilst NR2 subunits contain the glutamate binding site [6]. The NR1-NR2 diheterotetrameric constellation is the most common in the CNS and is sensitized by membrane depolarization, exhibit channel blockade by Mg^2+^ and allows high level, mainly Ca^2+^-selective conductance, although these parameters vary depending on the NR2 subtype [6]. The third subunit (NR3), discovered in the developing brain [7,8], does not bind glutamate, but similarly to NR1 contains a glycine binding site. Subsequent functional studies concluded that the rare variant of the NMDARs consisting of NR1-NR3 subunits are glycine gated, excitatory ion channels, that are unresponsive to glutamate or NMDA and insensitive to membrane depolarization as they do not have Mg^2+^-blockade [9]. In addition, Ca^2+^-influx generated by channels consisting of NR1-NR3 subunits has a smaller amplitude in comparison to conventional NMDARs and probably leads to intracellular signals others than those evoked by activation of NR1-NR2 channels [10].

Emerging evidence revealed the presence of glutamate receptors in various tissues other than the brain [11] and functional receptors have been reported (over)expressed in various neoplastic cells (gastric, colorectal, hepatocellular, prostate, lung, breast, ovarian cancers, etc.) [12]. Cutaneous melanoma only makes 3% of all skin tumors, but is responsible for 65% of skin-tumor-related deaths [13]. High mortality rates and increasing incidence highlight [14] the importance of understanding melanocyte and melanoma biology, and glutamatergic signalling seems to be a significant part of that. Melanoma cells can release elevated amounts of glutamate through a possible autocrine loop [15] and wide range of data has been gathered on mGluRs in this tumor type [2]. On the other hand, less is known about iGluRs in melanoma cells and melanocytes, albeit a recent study showed in vitro and in vivo that iGluR inhibition by MK-801 resulted in decrease of migration and loss of tumor growth [16]. In terms of NMDARs and their subunits, mutations frequently appear in the NR2A gene, *GRIN2A* [17], correlating with decreased survival of melanoma patients [18]. Furthermore, *GRIN2A* was shown to be a tumor suppressor in melanoma as loss of its activity resulted in uncontrolled cell proliferation [19]. NMDARs are also present on melanocytes [20] and regulate cell morphology and melanosome transfer [21]. Despite the NR2(A) subunit having been more often in the spotlight in melanoma cells and melanocytes, functions of the NR1 and NR3 subunits in this context remain elusive.

The NR1 subunit has a particular molecular attribute, insofar as it may contain a nuclear localization signal (NLS). It was demonstrated that the NR1-1a splice variant bears a functionally relevant intracellular domain: a bipartite NLS close to the C terminus, in the C1 cassette [22]. The NLS is a sequence of basic amino acids that may be on the surface of proteins allowing them to bind importins, thus permitting translocation to the nucleus. Unusual subcellular localization may give rise to novel functions, especially in the pathophysiological context. However, it is not clear whether NR1 undergoes regulated intramembrane proteolysis or appears in full sequence in the nucleus. Besides NR1, no other NMDAR subunits have been shown to possess a NLS.

In our present study, we aimed to examine the detailed subcellular expression pattern of NMDAR subunits in melanoma cells and melanocytes. As the most striking novel observation, we found that cells all of the investigated melanoma cell lines possessed full size nuclear NR1 and NR3B, which phenomenon was not observed in normal human epidermal melanocytes (NHEM). Immunocytochemistry of the melanoma cells proved that NR1-NR3B form heteromer complexes in the nucleus of melanoma cells. This finding raises the possibility of the existence of a malignant transformation related, glycine driven nuclear Ca^2+^-signalling in melanoma cells and may open new perspectives in melanoma therapy. 

## 2. Results

### 2.1. Melanocytes and Melanoma Cells Express NMDAR Subunit mRNAs

**Figure 1:**
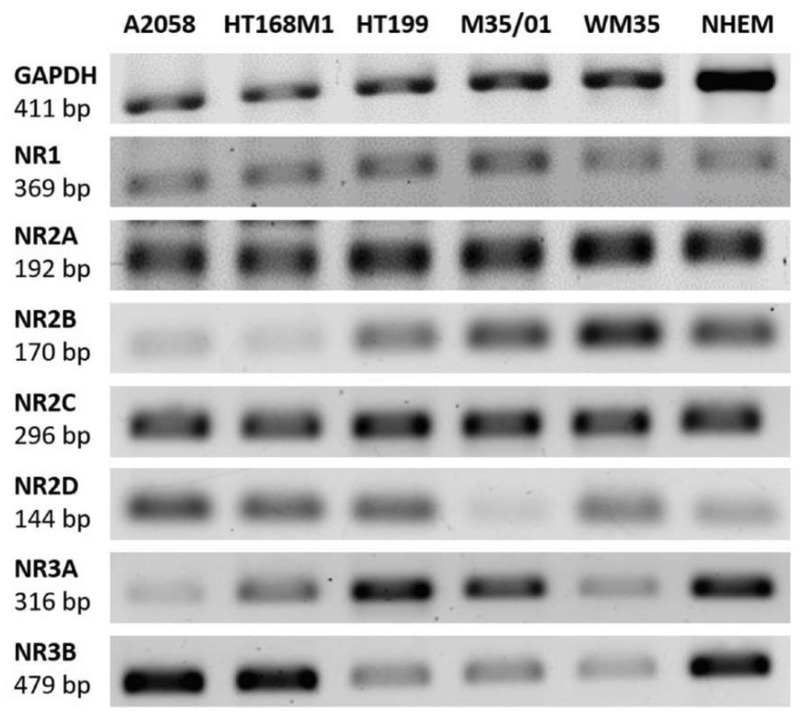
RT-PCR (reverse transcription followed by polymerase chain reaction) detection of NMDAR (*N*-methyl-d-aspartate receptor) subunit mRNA expression in melanoma cells and melanocytes. RT-PCRs proved the presence of the essential NR1, the widely expressed NR2 and also the rare NR3 subunits in A2058, HT169M1, HT199, M35/01 and WM35 melanoma cell lines, as well as in NHEMs (normal human epidermal melanocytes). GAPDH (Glycerol aldehyde phosphate dehydrogenase) mRNA expression served as internal control to the reaction. The positive control for NMDAR subunit detection was human brain tissue sample (see Supplementary Information Figure S1). Normalization of the relative optical density parameters of the subunit mRNA expressions to GAPDH is shown in a graphical format in the Supplementary Information Figure S2.

NMDA receptor subunits NR1 (*GRIN1*), NR2A (*GRIN2A*), NR2B (*GRIN2B*), NR2C (*GRIN2C*), NR2D (*GRIN2D*), NR3A (*GRIN3A*) and NR3B (*GRIN3B*) mRNA expression was examined by RT-PCR (reverse transcription followed by polymerase chain reaction) in five different melanoma cell lines (A2058, HT169M1, HT199, M35/01, WM35), as well as in melanocytes. In general, mRNAs of all NR subunits were detected either in the samples of melanoma cell lines or in that of melanocytes (Figure 1). Human brain sample was used as a positive control for detection of NMDAR subunit mRNAs (See Supplementary Information Figure S1). Glycerol aldehyde phosphate dehydrogenase (GAPDH) mRNA expression was the internal control of the reaction.

### 2.2. NR1 and NR3 Subunits Appear in Melanoma

**Figure 2:**
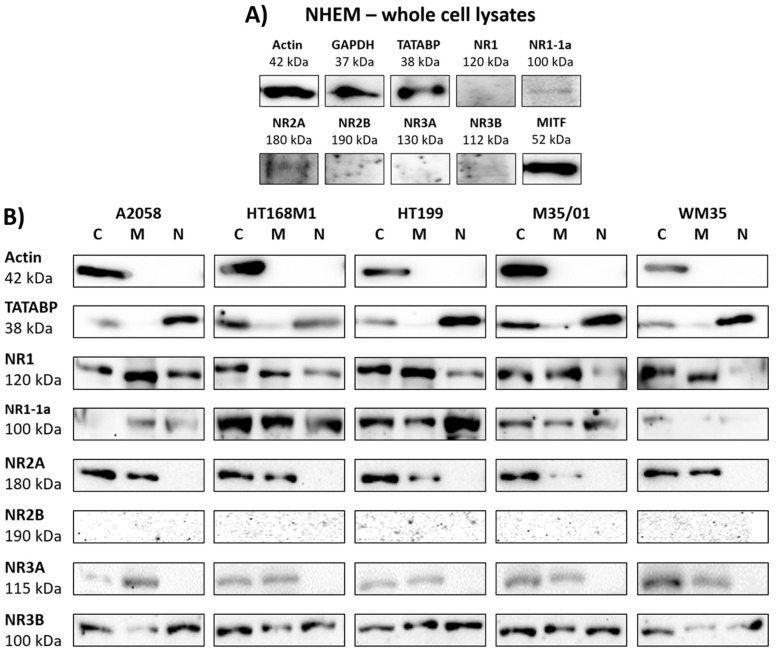
Western blot analysis gave detailed picture on NMDAR subunit protein expression. NR1-1a and NR2A subunits were expressed in melanocytes, while other subunits were undetectable by western blots (**A**). Fractionated melanoma samples revealed that NR1 and NR3B subunits are expressed in every cellular compartment (**B**). In each melanoma cell line NR2A and NR3A were present in the cytosol and membranes, but in the nucleus (**B**). NR2B was undetectable in each compartment (**B**). Immunoblot signals of NR1-1a were detected in the cytosol and membranes, but more importantly also in the nuclei of melanoma cells (**B**). Controls used in the experiments: MITF (microphthalmia associated transcription factor) for melanocyte differentiation control; GAPDH as an internal control for melanocyte whole cell lysates; actin as internal controls for the comparison of the cellular fractions; TATABP (TATA box binding protein) expression as internal control for nuclear fraction analysis. The positive control for detection of NMDAR subunit protein expression was human brain tissue lysate (see Supplementary Information Figure S3). Whole membrane pictures of western blots are presented as Supplementary Figure S4. Normalization of the relative optical density parameters of the protein subunit expressions to the respective control is shown in a graphical format in the Supplementary Information Figures S5–S7. (C = cytosolic, M = membrane, N = nuclear fractions of melanoma cells).

Western blot analysis of melanocyte whole cell lysates corroborated the presence of NR2A. Although the NR1-1a subunit showed immunoblot signals, NR1, NR2B, NR3A and NR3B subunits were under the level of detection in NHEM (Figure 2A). Microphthalmia-associated transcription factor (MITF) was used as a marker for melanocyte differentiation. Actin and GAPDH were used as internal controls for western blots of melanocyte whole cell lysates. 

Western blots of melanoma cellular fractions showed a detailed picture of NMDAR subunit expression in the cytosolic, nuclear and membrane compartments (Figure 2B). There was no difference found in the NMDAR subunit protein expression patterns of the radial growth phase (RGP) melanoma derived WM35 and the other, metastasizing melanoma-derived A2058, HT168M1, HT199 and M35/01 cell lines. The expression of NR1, NR2A, NR3A and NR3B subunits was confirmed in the cytosol and membranes of melanoma cells (Figure 2B). Immunoblot signals of the NLS containing NR1-1a splice variant were also detected. We found evidence of the presence of NR1, NR1-1a and NR3B subunits in the nuclei of melanoma cells, while NR2A and NR3A subunits were completely absent in the nuclear fractions (Figure 2B). NR2B signals remained undetectable in any of the examined cellular fractions, which confirmed lack or extremely weak protein levels of NR2B in melanoma cells. Multiple controls were used to prove clarity of fractionated samples. Actin was the internal control for the comparison of the cytosolic fraction to the membrane and to the nuclear fraction. TATA box binding protein (TATABP) was an internal control for nuclear fraction analysis. Human brain lysate was used as positive control for detection of NMDAR subunit protein expression (see Figure S3 in Supplementary Information).

### 2.3. Unusual Nuclear Colocalization of Subunits in Cultured Melanoma Cells, But Not in Melanocytes

**Figure 3:**
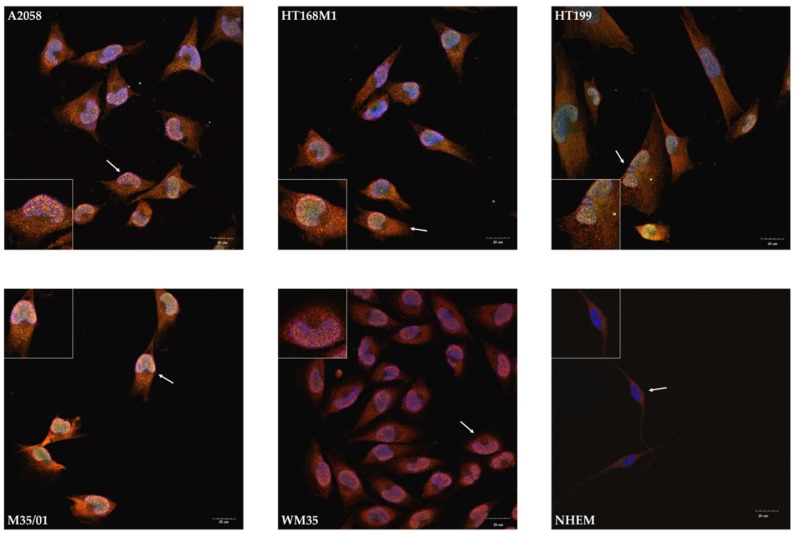
Immunocytochemistry demonstrating colocalization of NR1 and NR3B in the nuclei of melanoma cells, but not in the cell membrane in melanocytes. Confocal microscopic evaluation revealed that NR1 (red) was present in the cytosol of every melanoma cell line, while NR3B (green) showed weaker and less diffuse signals in the cytoplasm. Unambiguous plasma membrane immunofluorescent signals were not visible, but NR1 and NR3B colocalized inside the nuclei of melanoma cells. NR1 and NR3B colocalization was absent or undetected in the nuclei of NHEM. That 1-μm thick optical section was selected for presentation which specifically went through the majority of nuclei to show that all nuclei were immunopositive. One representative cell pointed by **white arrow** was selected for the inserts at each part of the panel. Scale bar: 20 μm. Positive controls for the secondary antibodies and special controls for the primary antibodies were also performed (see Supplementary Information Figure S8A,B).

**Figure 4:**
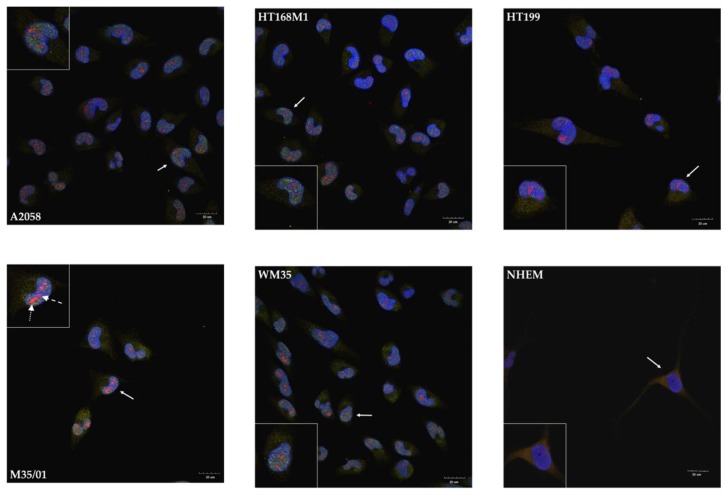
Immunocytochemistry demonstrating nuclear colocalization of NR1-1a and NR3B in melanoma cells. Confocal microscopic analysis demonstrated that NR1-1a (red) shows similar nuclear colocalization in melanoma cells with NR3B (green) as NR1. Nuclei of NHEM showed very weak signals of NR1-1a. NR1-1a was present in the cytoplasmic region of NHEM but unequivocal colocalization with NR3B was not detected at any cellular compartment. As an example, two extra arrows are used in the insert that shows M35/01 cells: the dashed arrow points at an area where the immunofluorescent signal of NR1-1a (red) colocalizes with DAPI (blue), resulting in purple tone whereas the spotted arrow points at an area where NR1-1a colocalizes with DAPI and NR3B (green), resulting in orange colour. Photomicrographs of 1 μm thick optical sections passing through the majority of nuclei in the area of the interest are shown. One representative cell pointed by **white arrow** with numerous (nuclear) colocalizing signals was selected on each picture and presented in the insert. Scale bar: 20 μm. Positive controls for the secondary antibodies and special controls for the primary antibodies were also performed (See Supplementary Information Figure S8A,B).

NR1-NR3B and NR1-1a-NR3B immunocytochemistry reactions were performed to reveal the subcellular (co)localization of the subunits. NR1 appeared in the cytosol of cells of every melanoma cell line, along with a less intense appearance of NR3B in the cytoplasm (Figure 3). Nevertheless, the nuclei showed remarkable patterns as NR1 and NR3B subunits colocalized in every investigated melanoma cell line (Figure 3). The prominent nuclear colocalization appeared diffusely inside the nuclei of melanoma cells rather than in the nuclear envelope or in perinuclear regions. We detected a similar expression pattern after the NR1-1a-NR3B immunocytochemistry reactions: the NLS containing NR1 splice variant specific antibody showed mostly nuclear localization and/or colocalizations with NR3B, while less cytoplasmic signals of NR1-1a were detected compared to NR1 (Figure 4). In another constellation of the experiments which was performed with an NR3B antibody produced in goat (a whole-membrane western blot picture confirming antibody specificity is shown as Figure S9), a more intense colocalization was observed, as seen in Supplementary Information Figure S10A,B. In contrast to these observations, melanocyte immunocytochemistry showed cytoplasmic localization of NR1 and NR3B subunits and confocal microscope revealed that the two subunits were undetectable in the nuclei (Figure 3). NR1-1a was also expressed in the cytoplasm of melanocytes but similarly to NR1, colocalization with NR3B could not be detected, although a very weak nuclear appearance of NR1-1a was observed (Figure 4).

## 3. Discussion

Here we presented proofs for the presence of full size NR1 and NR3B NMDAR subunits, forming heteromer complexes in the nuclei of human melanoma cells, raising the possibility of a glycine sensitive autonomous nuclear Ca^2+^-signalling of these cells. In the past 20 years, the nucleus has become a new field in the interpretation of cellular Ca^2+^-homeostasis as it emerged as a subcellular compartment to have its own Ca^2+^-related signalling [23,24,25]. Although the origin of Ca^2+^ is still a controversial question, several studies have been performed on isolated cell nuclei and showed that nucleoplasmic [Ca^2+^] changes are independent from the restricting second messengers (InsP_3_, cyclic ADP ribose) permeability of the nuclear membrane [26,27]. 

Cytosolic Ca^2+^ transients can ‘swamp’ Ca^2+^-changes of nuclear origin [28], making difficult to identify original nuclear transients. Nonetheless, certain observations showed that the nucleoplasmic reticulum and the nucleoplasm can generate autonomous Ca^2+^-signals [29]. In addition, other findings showed that the nuclear interior is rich in Ca^2+^-signalling components, such as inositol trisphosphate receptors (InsP_3_Rs), cyclic adenosine diphosphate ribose receptors (cADPRs), nicotinic acid adenine diphosphate (NAADP) receptors and even sarco/endoplasmic reticulum Ca^2+^-ATPase (SERCA) pumps and Na^+^/Ca^2+^-exchangers [23], supporting a concept of independent nuclear Ca^2+^-signalling.

Recent reviews reported that metabotropic receptors, especially a broad range of G protein coupled receptors are present in the nuclei of various cell types, such as cardiomyocytes, HEK293 and HeLa cells [30]. Evidence has been gathered on nuclear ionotropic receptors as well: proteomic analysis revealed R-type Ca^2+^-channels in the nuclear membrane, but K^+^ and Cl^–^ channels were also proved to be active by nuclear patch clamping [31,32].

Presence of functional metabotropic glutamate receptors (mGluR5) were reported in the nuclear membranes of HEK293 cells and cortical neurons. mGluR5 presumably activates nuclear phosphatidyl-inositol-phospholipase C (PtdIns-PLCs), resulting in InsP_3_ generation and consequent Ca^2+^-signals in the nucleoplasm [33,34]. But so far, no evidence of functional nuclear NMDARs or other nuclear ionotropic glutamate receptors has been published yet. 

Our results give pieces of evidence supporting the presence of ionotropic glutamate receptors in the nuclei of melanoma cells and afford valuable findings on NMDAR subunit expression in melanocytes. While a previous microarray analysis [20] revealed expression of NR2C subunit and confirmed absence of NR2B in melanocytes, our PCR results showed that all types of NMDAR subunits were present at mRNA level in NHEM. Nonetheless, western blots on NHEM could only prove the expression of NR1-1a and NR2A and very weak NR1 and NR3B signals were seen parallel to other subunits (NR2B, 2C and 3A) remaining undetectable. NR2A and NR3A were expressed in the cytosolic and membrane fractions of melanoma cells, but not in the nucleus, while NR2B was completely absent in melanoma cells, as in NHEM. This suggests that NR2 subunits do not play role in the possible nuclear NMDAR complex formation of melanoma cells. Therefore, detection of the essential NR1, and the unconventional NR3B subunits in every examined cellular compartments of melanoma cells may draw attention.

Confocal microscopic evaluation of immunocytochemistry reactions on melanoma cells demonstrated colocalization of NR1 and NR3B subunits suggesting the presence of molecular heteromers consisting of these subunits primarily inside the nuclei. We noticed spotted appearance of the colocalizing signals, which could be explained by the similar pattern in InsP_3_R expression [35]. That suggests NMDARs consisting of NR1 and NR3B subunits are likely to appear in the inner nuclear membrane, or in deep invaginations of the nuclear membrane or on the surface of nuclear Ca^2+^-storages. Colocalization of NR1 or NR1-1 with NR3B was not observed in the nuclei of melanocytes and remained weakly detectable in other parts of the cells. Taken together these findings further strengthen the possibility that NR1-1a and NR3B composed NMDARs locating in the nuclear compartment may be connected to malignancy.

Besides the presumable Ca^2+^-signalling role of these NMDAR complexes, another possible function (i.e., playing a direct role in transcription regulation) was also considered to explain nuclear presence of NMDAR subunits. We analysed if RNA, DNA or histone binding sequences are present either in NR1 or NR3B proteins by corresponding protein sequence databases (www.uniprot.org). As this screening carried out negative results we excluded the opportunity of an unusual function of these heteromers that is a role influencing transcriptional events or chromatin structure via direct molecular interactions with chromatin.

Conventional NMDARs are composed of two glycine binding NR1 subunits and two glutamate binding NR2 subunits each and are efficiently activated upon simultaneous binding of both glutamate and glycine. As neither NR1 nor NR3 subunits bind glutamate but glycine, these unorthodox NMDARs may function as alternative glycine receptors [36] which are permeable for Ca^2+^. Nonetheless, glutamate insensitivity and the fact that previous membrane depolarization is unnecessary for channel opening [9] make difficult to investigate the function of NR1-NR3 heteromers by pharmacological approaches. Of note, other electrophysiological properties of this channel constellation also differ from usual NR1-NR2 or NR1-NR2-NR3 channels in terms of Ca^2+^-currents [10]. 

As previously discussed, NR1-1a is the only splice variant of NR1 that contains NLS [22]. There has been no information published whether other NMDAR subunit splice variants possess NLS or not. As NLS in general is not a constant sequence that could be simply given, therefore, we decided to choose the NLSs described in NR1-1a [22] and based on sequence homology between human NR1 and NR3B. We matched the NLSs with the full-protein sequence of NR3B with the help of internet databases (www.uniprot.org). With this method, NLS-like motifs were found close to the intracellular C-terminus of NR3B (between amino acids 922 and 933), similar to NR1-1a. Therefore, we hypothesize the possibility of NR3B to have a functional NLS that could explain its nuclear localization. However, the NLS-suspicious area of NR3B needs thorough examination and functional confirmation [37]. 

Enhancing or modulating nuclear Ca^2+^-signalling could already be useful for treatments of neurodegenerative diseases, anxiety and post-traumatic stress disorders [38], moreover successful in vitro studies have reported the efficacy of NMDAR (and iGluR) antagonists on cancer and melanoma cells [39]. However, novel melanoma (and cancer) therapies involving nuclear Ca^2+^-signalling would still raise many questions, but the possibility is given for further examination and clarification as the nuclear Ca^2+^-signalling machinery and according to our results NMDARs are present in melanoma cells.

## 4. Materials and Methods

### 4.1. Melanoma and Melanocyte Cell Cultures

The human cutaneous melanoma cell line A2058 was derived from lymph node metastasis of amelanotic melanoma; HT168M1 was established from the spleen of HT168-injected immunosuppressed mice; HT199 was isolated from a metastasizing nodular melanoma; M35/01 was selected from a vertical growth phase (VGP) superficial spreading melanoma; and WM35 was derived from nonmetastasizing superficial spreading melanoma. A2058 and WM35 cell lines were acquired from ATCC (ATCC^®^ CRL-1661™, Manassas, VA, USA). HT168M1, HT199 and M35/01 cell lines were kind gifts of Andrea Ladányi (National Institute of Oncology, Budapest, Hungary). NHEM (PromoCell GmbH, Heidelberg, Germany) were isolated from the epidermis of juvenile or adult skin from different locations (face, breasts, abdomen, or thigh). Melanoma cells were cultured in RPMI-1640 culture medium (Sigma-Aldrich, St. Louis, MO, USA) supplemented with 10% FBS (Gibco, Gaithersburg, MD, USA), 4.1 g/L glucose, 2 mmol/L l-glutamine (Gibco), penicillin (100 units/mL) and streptomycin (100 µg/mL), and incubated at 37 °C, in a 5% CO_2_ atmosphere and 80% humidity to approximately 80% confluence. NHEM cell line was cultured in Melanocyte Medium (PromoCell GmbH) according to the instructions of the manufacturer. Every cell line was routinely screened for a possible *Mycoplasma* infection.

### 4.2. mRNA Expression Analysis Using Reverse Transcription Followed by PCR (RT-PCR)

After reaching the expected confluence melanoma and melanocyte cell cultures were washed three times with physiological NaCl, dissolved in TRIzol (Applied Biosystems, Foster City, CA, USA), and following addition of 20% chloroform (Sigma-Aldrich) samples were centrifuged at 10,000× *g* for 15 min at 4 °C. Samples were incubated in 500 μL RNase-free isopropanol at −20 °C for 1 h, then total RNA was dissolved in nuclease-free water (Promega, Madison, WI, USA) and stored at −70 °C. The assay mixture for reverse transcriptase (RT) reactions was composed of 2000 ng RNA, 2 μL 10× RT random primers; 0.8 μL 25× deoxynucleotide triphosphate (dNTP) Mix (100 mM); 50 units (1 μL) of MultiScribe™ RT in 2 µL 10× RT buffer (High Capacity RT kit; Applied Biosystems, Foster City, CA, USA). DNA was transcribed at 37 °C for 2 h.

Amplifications of specific cDNA sequences were carried out using specific primer pairs that were designed by Primer Premier 5.0 software (Premier Biosoft, Palo Alto, CA, USA) based on human nucleotide sequences published in GenBank and purchased from Integrated DNA Technologies, Inc. (IDT; Coralville, IA, USA). The specificity of custom-designed primer pairs was confirmed in silico by using the Primer-BLAST service of NCBI (Available online: http://www.ncbi.nlm.nih.gov/tools/primer-blast/). Nucleotide sequences of forward and reverse primers and reaction conditions are shown in Table 1. PCR reactions were carried out in a final volume of 21 μL containing 1 μL forward and 1 μL reverse primers (10 μM), 0.5 μL cDNA, 0.5 μL dNTP Mix (200 μM), and 0.625 unit (0.125 μL) of GoTaq^®^ DNA polymerase in 1× Green GoTaq^®^ Reaction Buffer (Promega) in a programmable thermal cycler (Labnet MultiGene™ 96-well Gradient Thermal Cycler; Labnet International, Edison, NJ, USA) with the following protocol: 2 min at 95 °C for initial denaturation followed by 35 repeated cycles of denaturation at 94 °C for 1 min, primer annealing for 1 min at an optimised temperature for each primer pair (see Table 1), and extension at 72 °C for 90 s. After the final cycle, further extension was allowed to proceed for another 10 min at 72 °C. PCR products were analysed using horizontal gel electrophoresis in 1.2% agarose gel containing ethidium bromide (Amresco Inc., Solon, OH, USA) at 120 V constant voltage. Signals were developed with a gel imaging system (Fluorchem E, Protein Simple, San Jose, CA, USA). Optical densities of signals were measured by using ImageJ 1.46R bundled with Java 1.8.0_112 freeware (https://imagej.nih.gov/ij/), and results were normalized to the internal control.

### 4.3. SDS-PAGE and Western Blot Analysis

Melanoma and melanocyte cell cultures were washed with physiological NaCl solution then harvested. After centrifugation at 2000× *g* for 10 min at room temperature, cell pellets were suspended in 100 μL of RIPA (Radio Immuno Precipitation Assay) homogenization buffer containing 150 mM NaCl, 1.0% NP40, 0.5% sodium deoxycholate, 50 mM Tris, 0.1% SDS (pH 8.0), supplemented with protein inhibitors as follows: aprotinin (10 μg/mL), 5 mM benzamidine, leupeptin (10 μg/mL), trypsin inhibitor (10 μg/mL), 1 mM PMSF, 5 mM EDTA, 1 mM EGTA, 8 mM Na-fluoride and 1 mM Na-orthovanadate. All components were purchased from Sigma-Aldrich. Samples were stored at −70 °C. 

Due to the availability of limited number of cells, melanocytes’ whole cell lysates were only used for further steps. NHEM suspensions were sonicated by pulsing burst for three times 30 s by 50 cycles using an ultrasonic homogeniser (Cole-Parmer, Vernon Hills, IL, USA). Melanoma cell fractions were separated as described below. 

Samples for SDS-PAGE (sodium-dodecyl-sulphate polyacrylamide gel electrophoresis) were prepared by adding Laemmli’s electrophoresis sample buffer (4% SDS, 10% 2-mercaptoethanol, 20% glycerol, 0.004% bromophenol blue, 0.125 mM Tris–HCl; pH 6.8) to cell lysates to set equal protein concentrations, and heated at 95 °C for 10 min. 20 μL of NHEM lysate (2 μg/μL final concentration), 20 μL of cytosolic fraction (0.5 μg/μL), 20 μL of nuclear fraction (0.5 μg/μL) and 20 μL of membrane fraction (0.5 μg/μL) samples of melanoma cells were separated by 7.5% SDS-PAGE gel for immunological detection of NMDAR subunit proteins. Separated proteins were transferred to nitrocellulose membranes (Bio-Rad Trans Blot Turbo Midi Nitrocellulose Transfer Packs) by using a Bio-Rad Trans-Blot Turbo system (Bio-Rad Laboratories, Hercules, CA, USA). After blocking in 5% nonfat dry milk in PBS for 1 h, membranes were incubated with primary antibodies overnight at 4 °C as shown in Table 2. After washing for 30 min in PBS with 1% Tween-20 (Amresco) (PBST (phosphate buffered saline supplemented with 1% Tween-20)), membranes were incubated with the HRP (horse radish peroxidase)-conjugated secondary antibodies, anti-rabbit or anti-mouse IgG (Bio-Rad Laboratories, Hercules, CA, USA) in 1:1500 dilution. Membranes were developed by enhanced chemiluminescence (Advansta Inc., Menlo Park, CA, USA) according to the instructions of the manufacturer. Signals were developed with a gel imaging system (Fluorchem E, Protein Simple, San Jose, CA, USA). Optical densities of western blot signals were measured by using ImageJ 1.46R bundled with Java 1.8.0_112 freeware (https://imagej.nih.gov/ij/), and results were normalized to the internal control.

### 4.4. Subcellular Fractionation of Melanoma Cells

Cytosolic, membrane and nuclear fractions of melanoma cells were separated. Samples used for cytosol and membrane isolation were sonicated, and then centrifuged at 50,000× *g* for 90 min at 4 °C. Supernatants containing cytosolic fractions were separated. Pellets were resuspended in 60 µL of 1% Triton X-100 (Sigma-Aldrich) in RIPA buffer and triturated on ice for 1 h. Afterwards, samples were centrifuged again at 50,000× *g* for 55 min at 4 °C. Supernatants containing membrane fractions were carefully separated.

For nuclear fraction isolation, melanoma cells were suspended in 1 mL buffer-A (10 mM HEPES, 1.5 mM MgCl_2_, 10 mM KCl, 0.1 mM EDTA, 0.1 mM EGTA, 1 mM DTT, supplemented with protein inhibitors as described above) and stored at −70 °C. Samples were not sonicated. After the addition of 50 μL Igepal CA-630 (Sigma-Aldrich) cells were homogenized by Dounce homogenizer. Samples were centrifuged at 770× *g* for 10 min at 4 °C and pellets were resuspended in 1 mL 2.2 mM sucrose-buffer-A solution. This was followed by a second step of centrifugation at 40,000× *g* for 90 min at 4 °C. Pellets were washed twice by 0.25 mM sucrose-buffer-A solution and suspended in 200 μL of the same solution.

### 4.5. Immunocytochemistry and Confocal Microscopy

Immunocytochemistry was performed on cells cultured on the surface of coverslips to visualize intracellular localizations of NR1, NR1-1a and NR3B. Cultures were fixed in 4% paraformaldehyde (Sigma-Aldrich) solution for 1 h and washed in distilled water. After rinsing in PBS (pH 7.4), nonspecific binding sites were blocked with PBS supplemented with 1% BSA (Amresco) for 30 min at 37 °C. After that samples were washed again three times in PBS, and cultures were incubated with rabbit polyclonal anti-NR3B antibody (Alomone Labs, Jerusalem, Israel), at a dilution of 1:50 in PBST, at 4 °C overnight. On the following day after washing three times with PBS, biotinylated goat anti-rabbit antibodies were added onto the samples (Vector Laboratories, Burlingame, CA, USA), at a dilution of 1:1000 in PBST, at room temperature for 2 h. After washing, cultures were incubated with monoclonal anti-NR1 antibody (Cell Signaling Technology, Danvers, MA, USA) or polyclonal anti-NR1-1a antibody (Merck-Millipore, Billerica, MA, USA), both produced in rabbit, at a dilution of 1:50 at 4 °C overnight. On the third day the biotinylated goat antibody was visualized with Streptavidin Alexa Fluor 488 conjugate, while the NR1/NR1-1a antibodies were visualized with anti-rabbit Alexa Fluor 555 secondary antibody (Life Technologies Corporation, Carlsbad, CA, USA) at a dilution of 1:1000 in PBST. Cultures were mounted in Vectashield mounting medium (Vector Laboratories, Peterborough, England) containing DAPI for nuclear DNA staining. Control experiments for these immunocytochemistry reactions are described in Supplementary Material and presented on Supplementary Figure S8A,B, S9, S10.

Immunocytochemistry reactions were repeated three times with NHEM and each melanoma cell line, and five recordings were examined with each constellation of the reaction.

Photomicrographs of the cells were taken with an Olympus FV3000 confocal microscope (Olympus Co., Tokyo, Japan) using a 60x PlanApo N oil-immersion objective (NA: 1.42) and FV31S-SW software (Olympus Co., Tokyo, Japan). Z image series of 1-μm optical thickness were recorded in sequential scan mode. For excitation 488 and 543 nm laser beams were used. The average pixel time was 4 μsec.

## Figures and Tables

**Table 1:** Nucleotide sequences, amplification sites, GenBank accession numbers, amplimer sizes and PCR reaction conditions for each primer pair are shown.

Gene	Primer	Nucleotide Sequence (5′→3′)	GenBank ID	Annealing Temperature	Amplimer Size (bp)
NR1 *(GRIN1)*	sense	CCA CGC TGA GTG ATG GGA CA (1568–1587)	NM_007327.3	56.5 °C	369
	antisense	GGT ACT TGA AGG GCT TGG AAA A (1936–1915)			
NR2A *(GRIN2A)*	sense	TGC CAC AAC GAG AAG AAC (2946–2963)	NM_001134407.2	57 °C	192
	antisense	GAT GGA GAA GAG CAA CCC (3137–3120)			
NR2B *(GRIN2B)*	sense	CAA GAG GCG TAA GCA GC (3413–3429)	NM_000834.3	55 °C	170
	antisense	CCA GGT AGA AGT CCC GTA G (3582–3564)			
NR2C *(GRIN2C)*	sense	ACG TCC ACG GCA TTG TCT (676–693)	NM_000835.4	61 °C	296
	antisense	GGA TGT CAT TCC CAT AAC CA (952–971)			
NR2D *(GRIN2D)*	sense	TGG CAA GCA CGG AAA GAA GAT C (1618–1639)	NM_000836.2	62.5 °C	144
	antisense	TCC ACG AAG GGG ACG GAG AAG T (1761–1740)			
NR3A *(GRIN3A)*	sense	ACA CAA AAC CCA CTT CCA ACA TCC (2107–2130)	NM_133445.2	58 °C	316
	antisense	TGC TCC ATA CTT TCC ATC CCC TAC (2422–2399)			
NR3B *(GRIN3B)*	sense	CGC AAG TGC TGC TAC GGC TAC (1436–1456)	NM_138690.2	61 °C	479
	antisense	ACG GTG CGT CTG AAG AGG ATG (1914–1894)			
GAPDH	sense	CCA GAA GAC TGT GGA TGG CC (740–759)	NM_002046.5	54 °C	411
	antisense	CTG TAG CCA AAT TCG TTG TC (1150–1131)			

**Table 2:** Tables of antibodies used in the experiments.

Antibody	Host Animal	Dilution for Western Blot	Dilution for Immunocytochemistry	Distributor, Cat. Num.
Anti-NR1	rabbit, monoclonal	1:250	1:50	Cell Signaling Technology, Danvers, MO, USA
				#5704S lot:2
Anti-NR1-1a	rabbit, polyclonal	1:200	1:50	Merck-Millipore, Billerica, MA, USA
				AB5046P
Anti-NR2A	rabbit, polyclonal	1:200	-	Cell Signaling Technology, Danvers, MO, USA
				#4205S lot:1
Anti-NR2B	rabbit, polyclonal	1:200	-	Cell Signaling Technology, Danvers, MO, USA
				#4207S lot:2
Anti-NR3A	rabbit, polyclonal	1:300	-	Merck-Millipore, Billerica, MA, USA 07-356
Anti-NR3A	rabbit, polyclonal	1:500	-	Alomone Labs, Jerusalem, Israel AGC-030
Anti-NR3B	rabbit, polyclonal	1:600	-	Abcam, Cambridge, UK
				ab35677-100
Anti-NR3B	rabbit, polyclonal	1:250	1:50	Alomone Labs, Jerusalem, Israel AGC-031
Anti-TATABP	mouse, monoclonal	1:500	-	Abcam, Cambridge, UK ab51841
Anti-GAPDH	rabbit, polyclonal	1:500	-	Abcam, Cambridge, UK Ab9485
Anti-β-Actin	mouse, monoclonal	1:10.000	-	Sigma-Aldrich, St. Louis, MO, USA A5441


## Supplementary Materials

Supplementary materials can be found at http://www.mdpi.com/1422-0067/19/7/1929/s1.

## Abbreviations

CNS	central nervous system
iGluR	ionotropic glutamate receptor
mGluR	metabotropic glutamate receptor
NMDA	*N*-methyl-d-aspartate
NMDAR	*N*-methyl-d-aspartate receptor
NLS	Nuclear localization signal
NHEM	normal human epidermal melanocytes
RT-PCR	reverse transcription followed by polymerase chain reaction
dNTP	deoxynucleotide triphosphate
SDS-PAGE	sodium-dodecyl-sulphate polyacrylamide gel electrophoresis
RIPA	radio immuno precipitation assay
EDTA	ethylene diamine tetra-acetic acid
EGTA	ethylene glycol tetra-acetic acid
PBS	phosphate buffered saline
PBST	phosphate buffered saline supplemented with 1% Tween-20
HRP	horse radish peroxidase
HEPES	hydroxyethyl-piperazine-ethanesulfonic acid
DTT	dithiothreitol
GAPDH	glycerine aldehyde phosphate dehydrogenase
MITF	microphthalmia associated transcription factor
TATABP	TATA box binding protein
InsP_3_R	inositol trisphosphate receptor
