# Supplementary material for: NR1 and NR3B Composed Intranuclear N-methyl-d-aspartate Receptor Complexes in Human Melanoma Cells
Source: Int J Mol Sci. 2018 Jun 30;19(7):1929. doi: 10.3390/ijms19071929 (PMC6073738; doi:10.3390/ijms19071929)
Supplement: Supplementary file 1 [file ijms-19-01929-s001.pdf]

International Journal of
Molecular Sciences

MDPI

Supplementary information to the manuscript of *"NR1 and NR3B composed N-methyl-D-aspartate receptor complexes in human melanoma cells"* by Tibor Hajdú *et al.*

1. NMDAR subunit primer testing in human brain for ideal mRNA expression profile

In our Manuscript it is stated that human brain sample was used as positive control for detection of NMDAR subunit mRNAs. To determine the ideal annealing temperature we performed gradient PCRs, results are shown in Supplementary Figure S1.

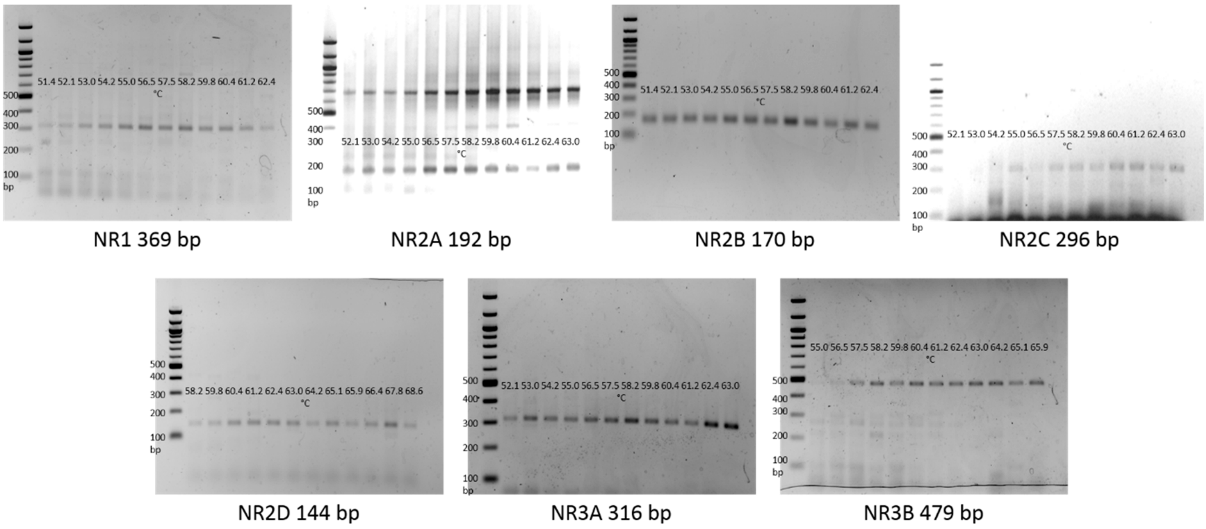

Supplementary Figure S1. mRNA expression pattern of NMDAR subunits in human brain sample. mRNA expression was detected by gradient RT-PCRs. Temperature values are indicated above each sample. Temperature intervals were determined according to the recommended annealing temperature of the sense and antisense primer.

Human brain samples were obtained from the Department of Forensic Medicine, University of Debrecen. The study was approved by the Ethics Committee of University of Debrecen, under licence number 3244-7/2011. Total RNA was isolated as described in the Materials and Methods section of the Manuscript. Reverse transcription followed by polymerase chain reaction (PCR) were performed in a programmable thermal cycler (Labnet MultiGene™ 96-well Gradient Thermal Cycler; Labnet International, Edison, NJ, USA) with the reaction settings described in the Manuscript. Nucleotide sequences of forward and reverse primers and reaction conditions are shown in Table 1 in the Manuscript. PCR products were analysed using a 1.2% ethidium bromide-containing agarose gel. Optical densities of signals were measured by using using ImageJ 1.46R bundled with Java 1.8.0_112 freeware (https://imagej.nih.gov/ij/), and results were normalised to the internal control.

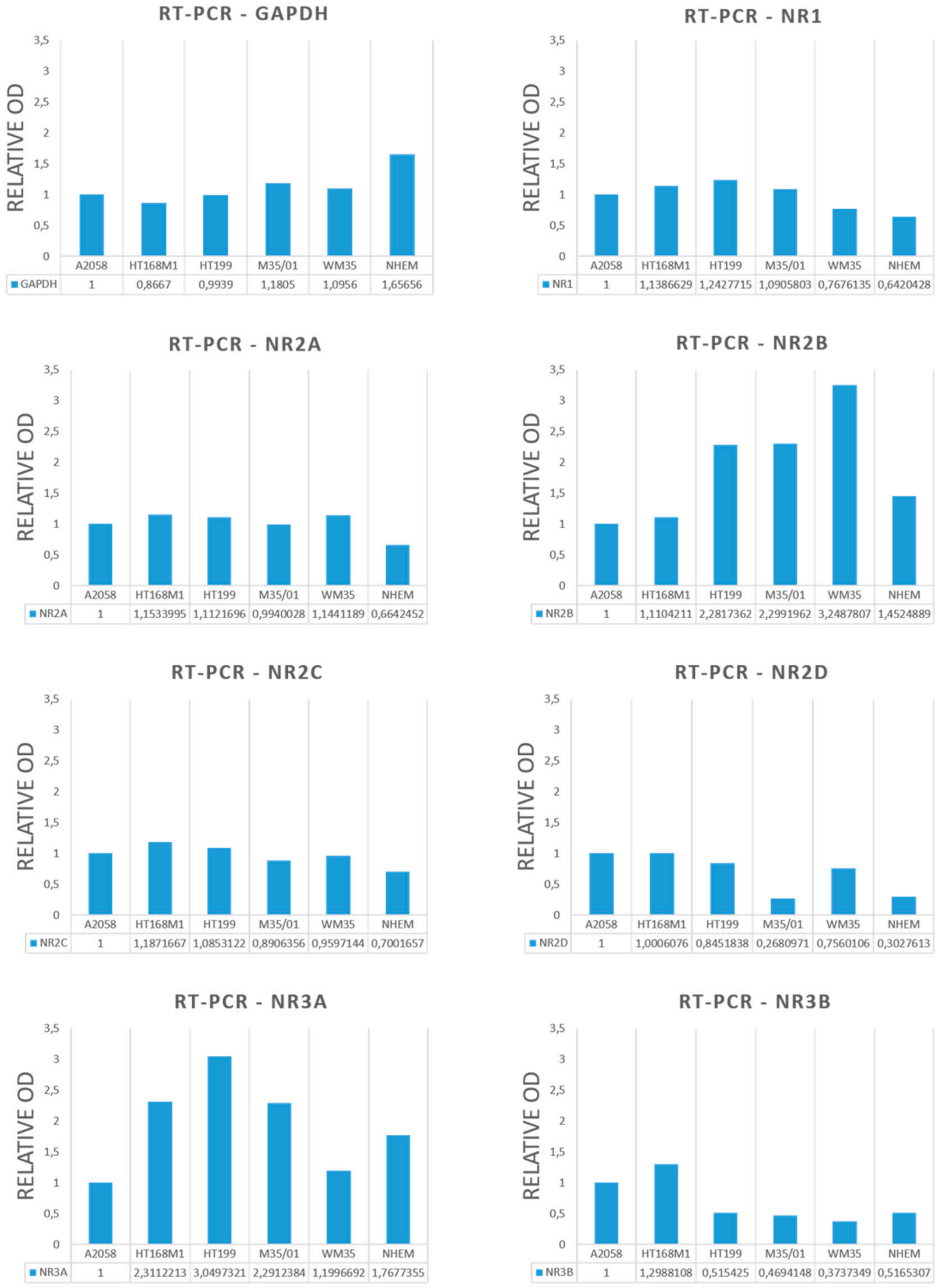

Supplementary Figure S2. Relative optical density (OD) of NMDAR subunit mRNA expression normalized to GAPDH (internal control) expression in the examined melanoma cell lines and NHEM. Only one sample of each cell line could be examined in a single gel electrophoresis, therefore one column represents the OD of one sample from the exactly same experimental circumstances.

2. Specificity control of NMDAR subunit antibodies in human brain lysate

As shown in Supplementary Figure S3., human brain lysate was used as positive control for NMDAR subunit protein expression. Specificity of the antibodies was tested by western blots before use. Whole membranes of western blots on fractionated melanoma samples are presented on

Supplementary Figure S4. Parameters of the antibodies used in the experiments are shown in Table 2 in the main text.

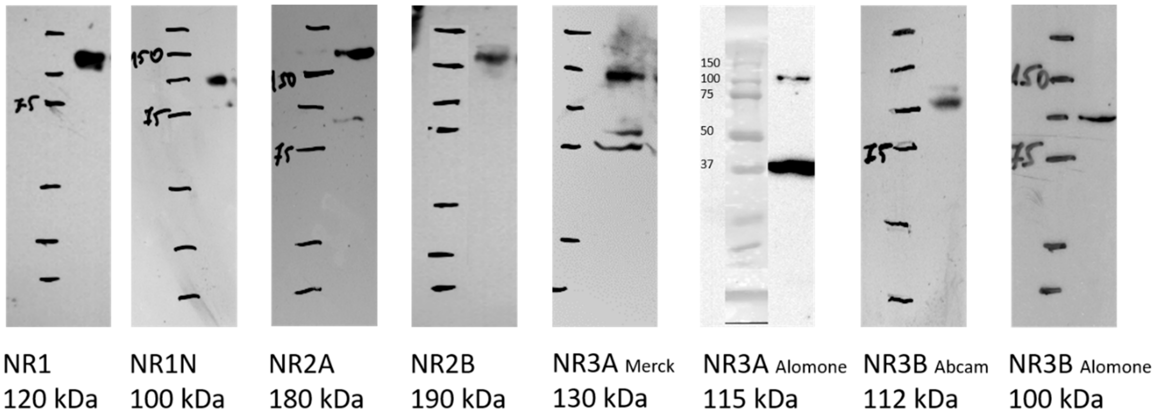

Supplementary Figure S3. NMDAR subunit protein expression profiles on human brain lysates served as positive controls for western blots and immunocytochemistry reactions on melanoma cells and melanocytes. Bio Rad dual colour Precision Plus Protein Standards were used as ladder, steps from bottom to top: 37, 50, 75, 100, 150, 250 kDa.

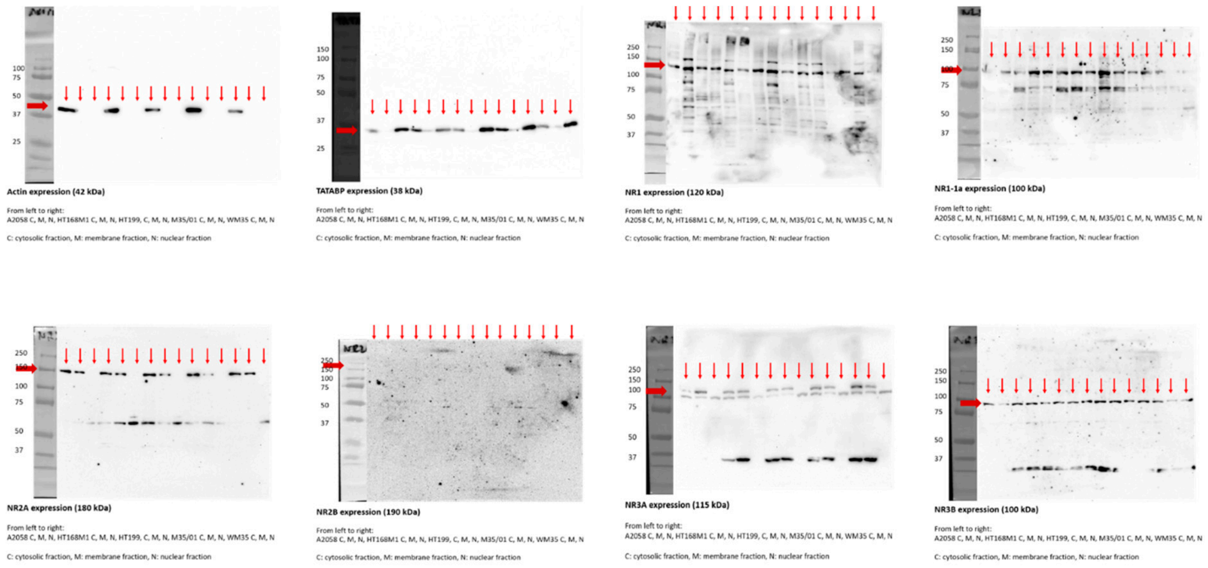

Supplementary Figure S4. NMDAR subunit and internal control (actin, TATABP) protein expression profiles by western blotting fractionated samples of melanoma cells (from left to right: A2058, HT168M1, HT199, M35/01 and WM35). C, M and N refer to the cytosolic, membrane and nuclear fractions of melanoma cells, respectively (marked by the red arrows for each cell line). Bio Rad dual colour Precision Plus Protein Standards were used as ladder, steps from bottom to top: 37, 50, 75, 100, 150, 250 kDa.

3. Quantification of NMDAR subunit expression in cellular fractions of melanoma cell lines

Western blots on cellular fractions (cytosol, membrane, nucleus) of melanoma cells were performed as described in the main text. Optical densities of western blot signals were measured by using using ImageJ 1.46R bundled with Java 1.8.0_112 freeware (https://imagej.nih.gov/ij/), and normalization against the control in the cytosolic and nuclear fraction is shown in graphical form in Supplementary Figures S4 and S6. Relative OD parameters of the subunit expression in the membrane fraction is presented in Supplementary Figure S5.

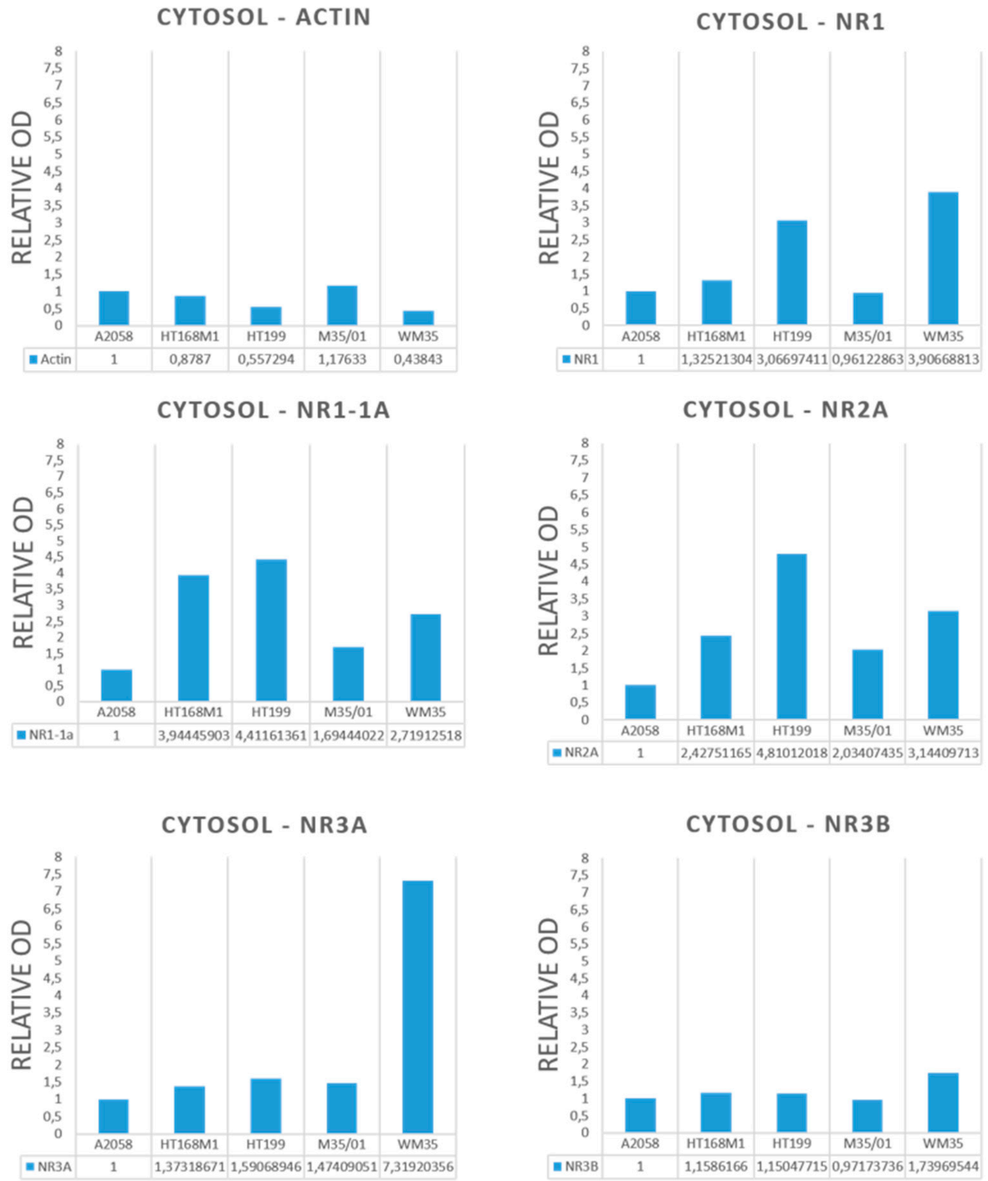

Supplementary Figure S5. Relative optical density (OD) of NMDAR subunit protein expression normalized to actin (internal control) expression in the cytosolic fraction of the examined melanoma cells. Only one sample of each subcellular fraction of each cell line could be examined in a single gel electrophoresis, therefore one column represents the OD of one sample from the exactly same experimental circumstances. Only those subunit expressions were quantified that showed immunoblot signals; that is the reason why NR2B expression is not represented on a diagram.

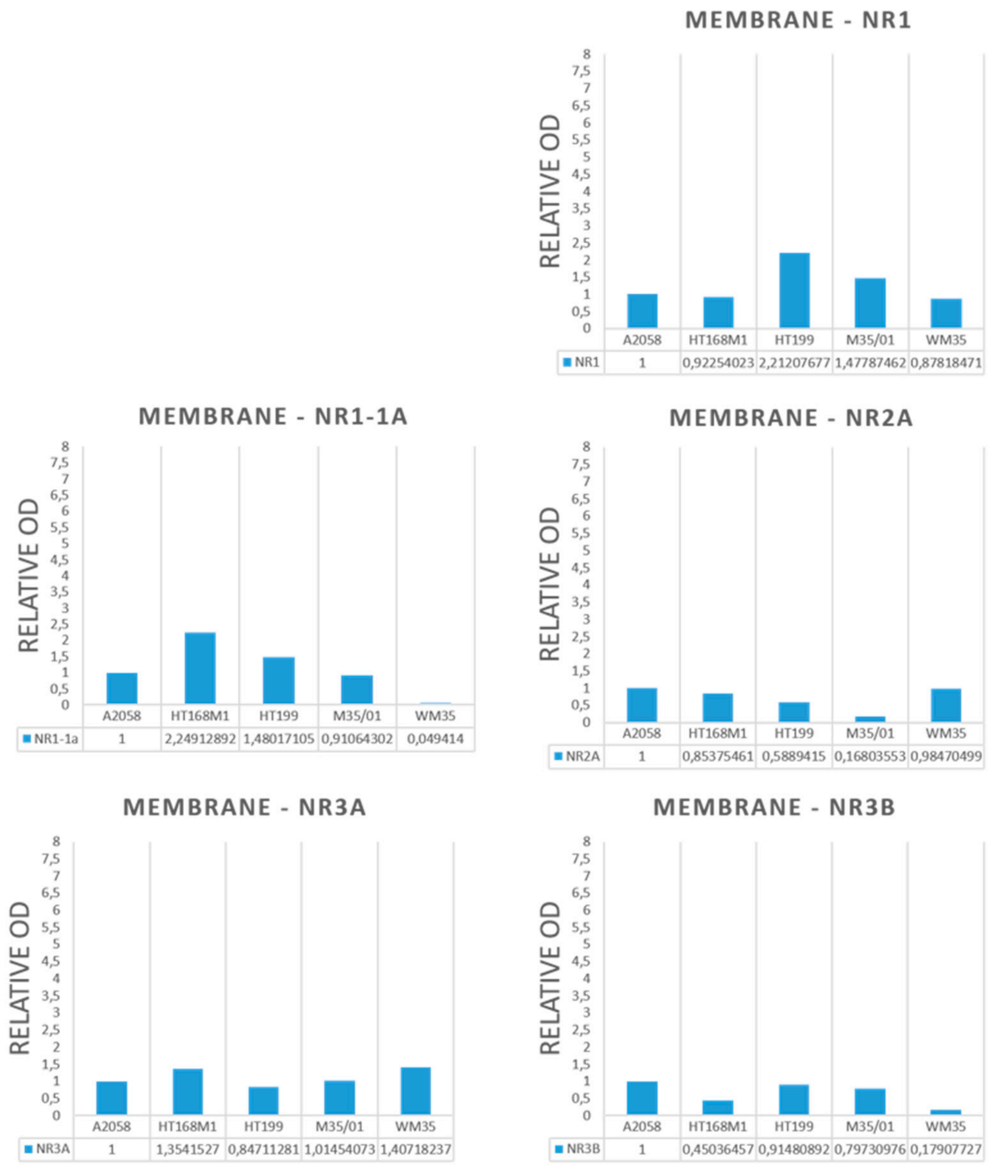

Supplementary Figure S6. Relative optical density (OD) of NMDAR subunit protein expression in the membrane fraction of the examined melanoma cells. Only one sample of each subcellular fraction of each cell line could be examined in a single gel electrophoresis, therefore one column represents the OD of one sample from the exactly same experimental circumstances. Lacking expression of actin (internal control) was intended to be the control of successful fractionation of the membrane fraction; however, NMDAR subunit expression could not be normalized on that. Only those subunit expressions were quantified that showed immunoblot signals; that is the reason why NR2B and actin expressions are not represented on a diagram.

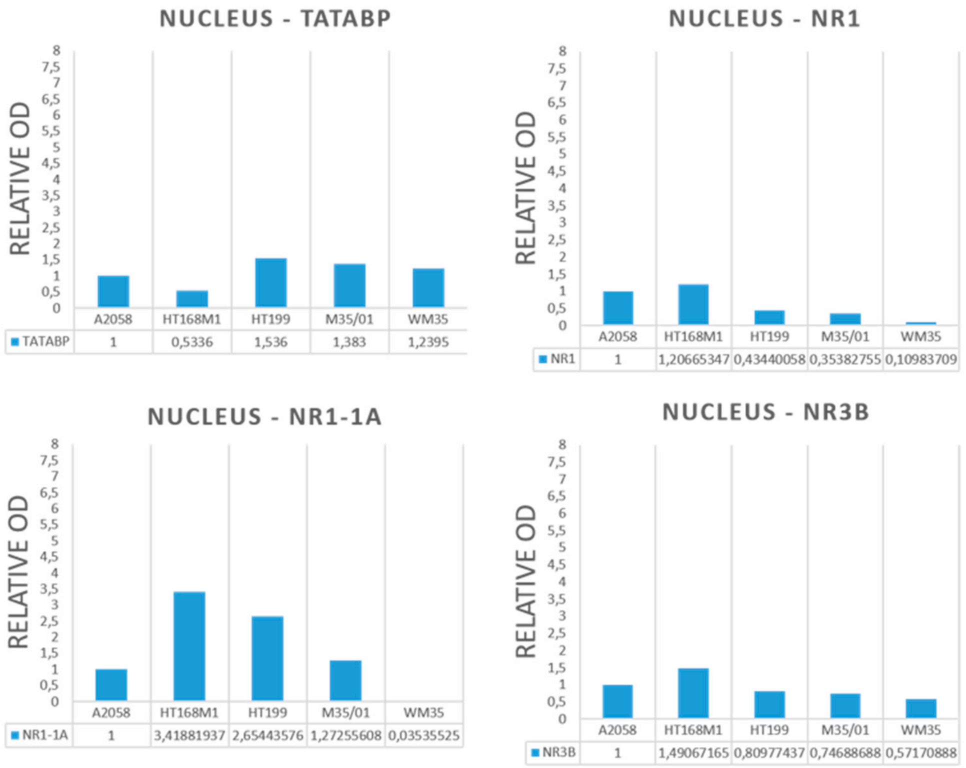

Supplementary Figure S7. Relative optical density (OD) of NMDAR subunit protein expression normalized to TATABP (internal control) expression in the nuclear fraction of the examined melanoma cells. Only one sample of each subcellular fraction of each cell line could be examined in a single gel electrophoresis, therefore one column represents the OD of one sample from the exactly same experimental circumstances. Only those subunit expressions were quantified that showed immunoblot signals; that is the reason why NR2A, NR2B and NR3A expressions are not presented on a diagram.

4. Controls of NR1-NR3B and NR1-1a-NR3B immunocytochemistry reactions

To investigate if we can confirm colocalization of NR1(-1a) and NR3B subunits in the nucleus we performed immunocytochemistry reactions. For these experiments we needed to perform a special control as both primary antibodies were produced in the same species. In this control we only applied the NR3B antibody, and omitted NR1 or NR1-1a antibodies. Then the biotinylated secondary antibody was added to the samples to bind to NR3B. After the addition of the fluorochrome labelled antibodies (Streptavidin Alexa 488 conjugate (green colour) and Alexa Fluor 555 (red colour)) we examined whether colocalizing fluorescent signals appeared in our samples. We expected that the avidin-biotin complex that binds to NR3B occupies the surface for the attachment of other secondary antibodies. Since we detected only green signals but no red, our colocalizing signals were judged to be verified and excluded the possibility of binding of the two different fluorochrome labelled antibodies to the same primary antibody, thereby getting a false positive result for colocalization. Results of this reaction are presented in Supplementary Figure S8A.

Negative control reactions of the fluorochrome labelled antibodies showed that in absence of the primary antibodies and the biotinylated secondary antibody immunofluorescent signals could not be detected. Results are shown in Supplementary Figure S8B.

Immunocytochemistry reactions were repeated three times with NHEM and each melanoma cell line, and five recordings were examined with each constellation of the reaction.

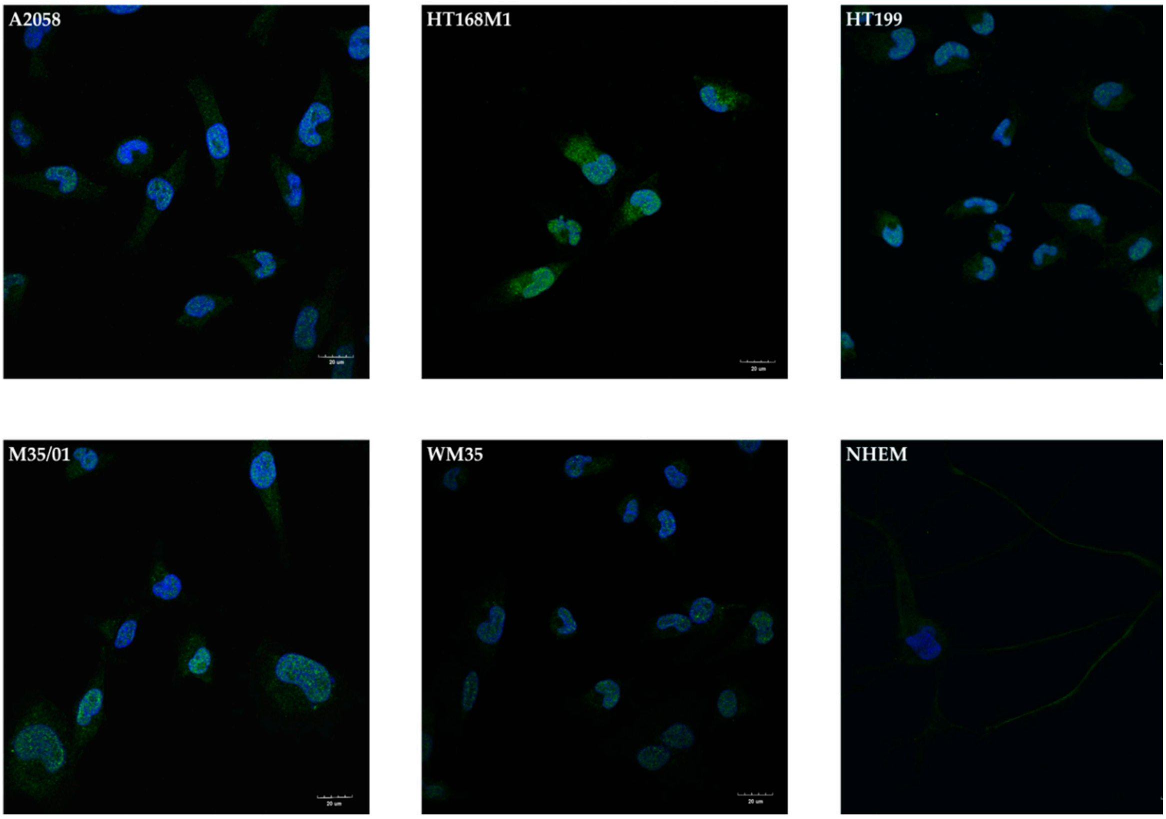

(**A**)

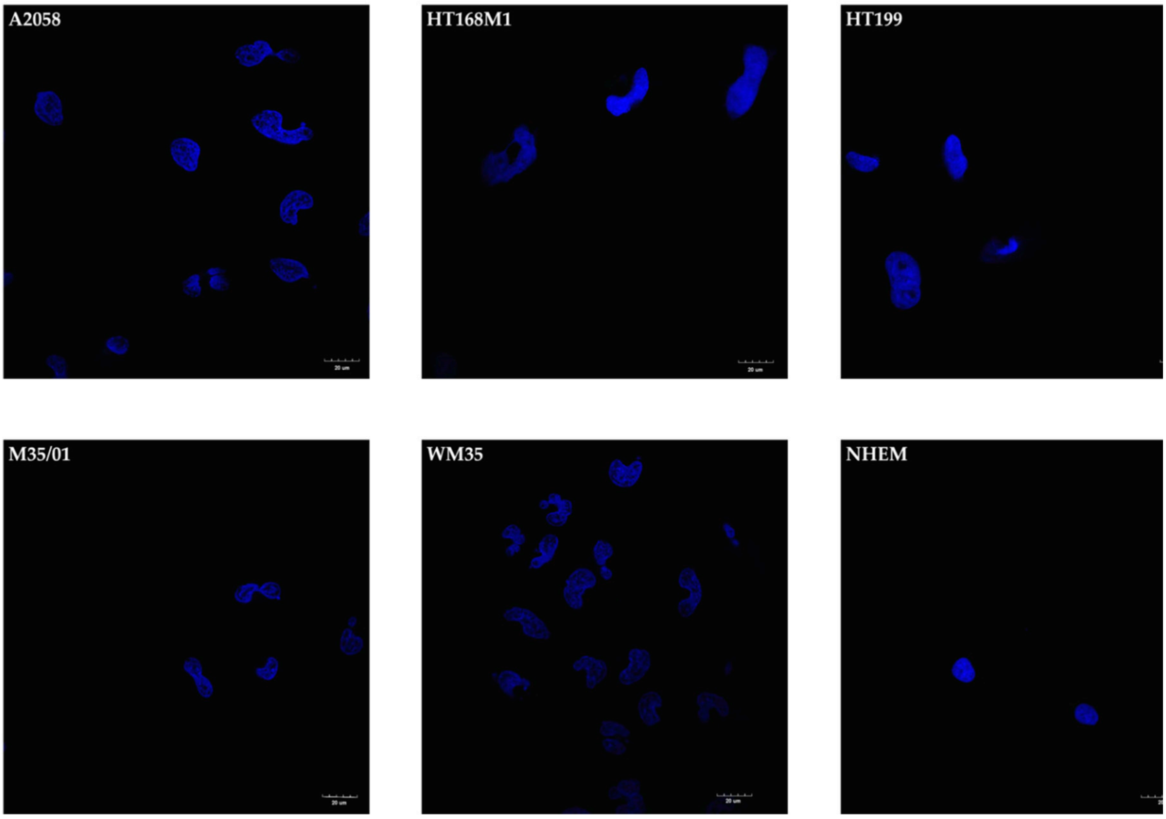

(**B**)

Supplementary Figure S8. Immunocytochemistry reactions demonstrating results of the special control experiments (A) and the negative control reactions of the fluorochrome labelled antibodies. After confocal microscopic analysis a 1-µm thick optical section was selected for presentation that went through the majority of nuclei to determine whether the nuclei were immunopositive. Scale bar: 20 µm.

Other controls were also performed on A2058, WM35 and NHEM cell lines to make it fully clear that the constellation used in previous experiments is reliable. Anti-NR3B antibody produced in goat (ab106808, Abcam, Cambridge, UK) was obtained and reactions were examined with NR1 and NR1-1a. Before use for immunocytochemistry reactions, this anti-NR3B antibody was tested by western blots on human brain samples as seen in Supplementary Figure S9. The anti-NR3B antibody was used at a dilution of 1:250 in milk-PBS for western blots and at a dilution of 1:50 in PBST for immunocytochemistry reactions. The anti-NR3B antibody was visualized with anti-goat Alexa Fluor 488 antibody produced in donkey ((Life Technologies Corporation, Carlsbad, CA, USA) at a dilution of 1:1000 in PBST. Results of the reactions are presented in Supplementary Figure S10A–D.

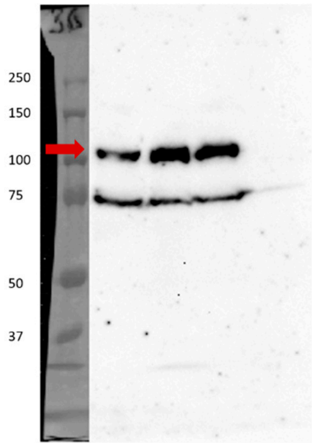

Supplementary Figure S9. Expression profile of the anti-NR3B antibody produced in goat (ab106808) on three different human brain lysates served as positive control for immunocytochemistry reactions on melanoma cells and melanocytes. Expected band size: 113 kDa, as the arrow shows. Bio Rad dual colour Precision Plus Protein Standards were used as ladder, steps from bottom to top: 37, 50, 75, 100, 150, 250 kDa.

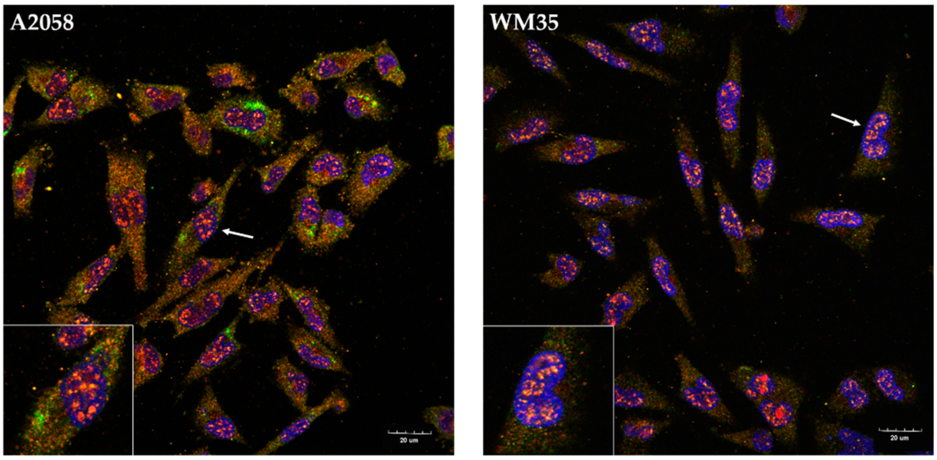

(**A**)

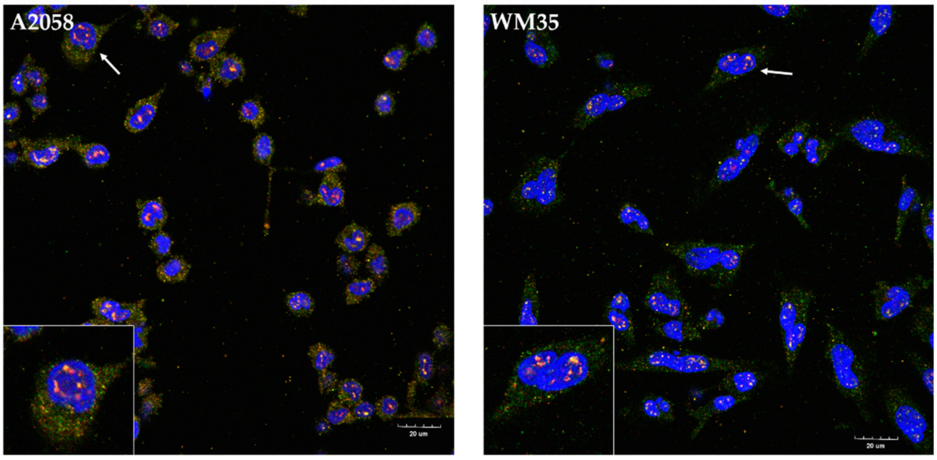

(**B**)

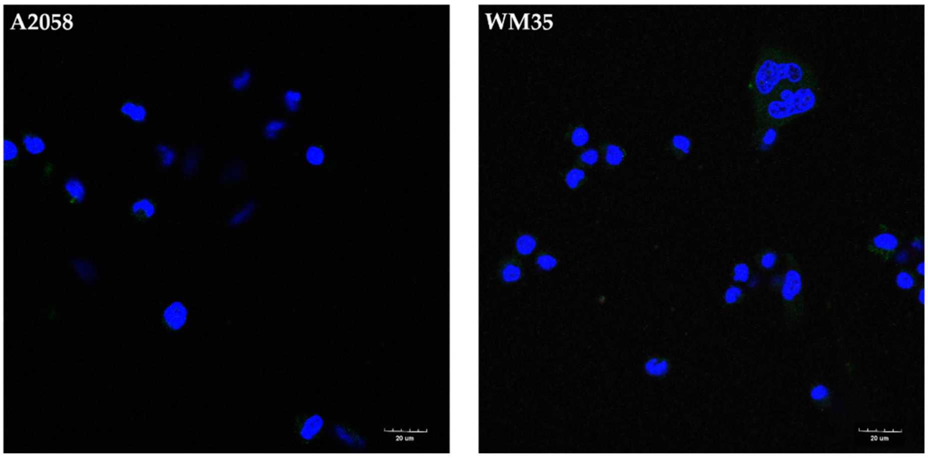

(**C**)

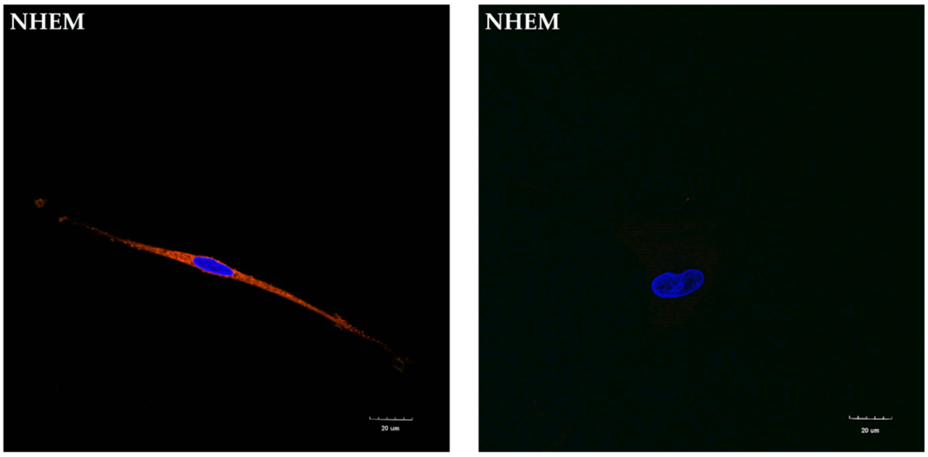

(**D**)

Supplementary Figure S10. Confocal microscopic analysis revealed that NR1 (red, primary antibody produced in rabbit) colocalizes with NR3B (green, primary antibody produced in goat) inside the nuclei of A2058 and WM35 cells (A). NR1-1a (red, primary antibody produced in rabbit) also showed similar but less nuclear colocalization in melanoma cells with NR3B (green) (B). On Supplementary Figure C the negative control reactions of the fluorochrome labelled antibodies are presented (anti-rabbit Alexa Fluor 555 and anti-goat Alexa Fluor 488 are only added). Colocalization of NR1 and NR3B was not detected in the nuclei of NHEM, but immunopositive signals were present in the cytoplasm (left picture of Supplementary Figure D). Reactions with NR1-1a were not performed in NHEM. The right picture of Supplementary Figure D shows the negative control reactions of the fluorochrome labelled antibodies. Photomicrographs of 1 μm thick optical sections passing through the majority of nuclei in the area of the interest are shown. One representative cell with numerous

(nuclear) colocalizing signals was selected on Supplementary Figure 10A and B and presented in the insert. Scale bar: 20 μm.

Photomicrographs of the cells were taken with an Olympus FV3000 confocal microscope (Olympus Co., Tokyo, Japan) using a 60x PlanApo N oil-immersion objective (NA: 1.42) and FV31S-SW software (Olympus Co., Tokyo, Japan). Z image series of 1-μm optical thickness were recorded in sequential scan mode. For excitation, 488 and 543 nm laser beams were used. The average pixel time was 4 μsec.
